# Potential methanol toxicity and the importance of using a standardised alcohol-based hand rub formulation in the era of COVID-19

**DOI:** 10.1186/s13756-020-00788-5

**Published:** 2020-08-08

**Authors:** K. Dear, L. Grayson, R. Nixon

**Affiliations:** 1 Occupational Dermatology Research and Education Centre, Skin Health Institute, Level 1/80 Drummond Street, Melbourne, Australia; 2 University of Melbourne, Parkville, Victoria, Australia; 3 Australia and University of Melbourne, Parkville, Victoria, Australia

**Keywords:** Alcohol-based-hand-rubs, Methanol toxicity, SARS-CoV-2

## Abstract

**Objectives:**

Hand sanitisers are urgently needed in the time of COVID-19, and as a result of shortages, some people have resorted to making their own formulations, including the repurposing of distilleries. We wish to highlight the importance of those producing hand sanitisers to avoid methylated spirits containing methanol and to follow WHO recommended formulations.

**Methods:**

We explore and discuss reports of methanol toxicity through ingestion and transdermal absorption. We discuss the WHO formulations and explain the rationale behind the chosen ingredients.

**Short conclusion:**

We advise those producing hand sanitisers to follow WHO recommended formulations, and advise those producing hand sanitisers using methylated spirits, to avoid formulations which contain methanol.

## Main text

The COVID-19 (SARS-CoV-2) pandemic, declared by the World Health Organization (WHO) on 11th March 2020 has led to a global shortage in commercially available alcohol-based hand rubs (ABHRs). Whilst hand washing with soap and water remains the effective method to reduce transmission of the virus, ABHRs provide a convenient, effective and relatively low cost alternative worldwide [1].

Several countries including the United States of America (USA), Australia and the United Kingdom (UK) have relaxed legislation to make it easier for local businesses to rapidly produce ABHRs [2–4]. The Australian Therapeutic Goods Administration (TGA) currently allows for hand sanitizer to be produced without the requirement of TGA approval or notification, provided one of the two formulations developed by the WHO are used [3]. In the UK and USA, local businesses are allowed to produce hand sanitizer adhering to WHO formulations, however the alcohol used must be denatured prior to production of the ABHR [2, 4]. Consequently, there has been a surge in production of large quantities of ABHR by various businesses, including pharmacies and alcohol distilleries. The magnitude and speed in response is commendable in this era of unprecedented demand, but may also carry some potential risks.

The WHO’s recommended formulations utilise either ethanol 80% v/v (Formulation 1) or isopropyl alcohol 75% v/v (Formulation 2) each with glycerol 1.45% v/v, hydrogen peroxide (H_2_O_2_) 0.125% v/v and sterile distilled or boiled cold water [5]. Ethanol and isopropyl alcohol are chosen due to their marked viricidal effect by denaturing proteins and inactivating enveloped SARS-CoV, MERS-CoV - and more recently both formulations have been shown to be effective against SARS-CoV-2 [6, 7]. Benzalkonium chloride, an antiseptic used in some hand sanitisers, appears to have less reliable activity against coronaviruses than either ethanol or isopropyl alcohol and for this reason is not recommended first line for use against SARS-CoV-2 [8]. The low concentration of hydrogen peroxide within the WHO formulations is intended to eliminate any bacterial spores present in the formulation and is not considered part of the antisepsis. Glycerol is a safe, relatively inexpensive humectant that acts as an emollient to increase the tolerability of the product and protect skin from contact dermatitis with repeated use.

Unsurprisingly, the shortage of commercially available ABHRs has also led to a multitude of home ‘Do-It-Yourself” (DIY) hand sanitiser recipes circulating online. Several of these formulas use unvalidated methods and are from non-qualified sources. Of potential concern is where ‘methylated spirits’ (also known as denatured alcohol) is substituted for ethanol or isopropyl alcohol. Whilst in recent years in Australia, the formulation of methylated spirits has largely changed from including methanol (wood alcohol) to other safer denaturants such as denatonium benzoate, there are still some formulations of methylated spirits containing methanol available for purchase - admittedly often at a low concentration of 2% (e.g. ‘Gold Cross™). However, a recent Australian alert issued earlier this year described a patient who presented with methanol toxicity having ingested the contents of methylated spirits which was found to contain more than 60% methanol [9]. Worldwide, particularly in low income settings, methylated spirits products frequently contain methanol and are readily available [10]. Other regions such as the UK also permit methanol to be used in some trade-specific formulations of denatured alcohol [11]. In the past month in Dubai, six ABHRs of 102 tested have been removed from the market because of the presence of methanol [12].

Methanol toxicity as a result of ingestion, as well as from inhalation and transdermal absorption is not a new entity. Perhaps the most tragic reminder of the toxic effects of methanol can be seen in a recent poisoning epidemic in Iran, where at least 700 deaths occurred and over 2000 people became severely toxic after ingesting methanol in illegal alcoholic beverages in a bid to ‘prevent’ Covid-19 [13, 14]. Furthermore, there have been reports of methanol toxicity caused by ingestion of ABHRs as well as chronic methanol toxicity from transdermal absorption of these products, where the methanol content was not disclosed [15, 16]. While all the alcohols used in ABHRs may be toxic following ingestion, only methanol appears to be toxic following dermal absorption via these products [15, 17, 18]. The toxic effects of methanol compared with isopropyl alcohol and ethyl alcohol are summarised in Table 1.

**Table 1:** Toxic effects of methanol, isopropyl alcohol and ethyl alcohol

Alcohol	Dermal absorption from use of ABHRs	Toxic metabolite	Toxic effects
Methanol	Can occur with toxic effects [15].	Formic acid	Nausea Vomiting Abdominal pain Central nervous system (CNS) depression Visual disturbance Increased osmolar gap with metabolic acidosis [19].
Isopropyl alcohol	Can occur but not to toxic levels [20–22].	Acetone	Inebriation Gastrointestinal mucosal irritation (abdominal pain, vomiting, diarrhoea, haematemesis) Profound CNS depression (headache, dizziness, stupor/coma) Increased osmolar gap but no metabolic acidosis [19].
Ethanol	Can occur but not to toxic levels [21–24].	Acetaldehyde	Inebriation Gastrointestinal mucosal irritation (vomiting, haematemesis, diarrhoea) Hypoglycaemia CNS depression [25].

In light of the well-recognised dangers of methanol, and the absence of any precise legislation prohibiting its presence in methylated spirits, we conclude that methylated spirits should not be used in ABHRs, not least for the risk of toxicity by ingestion as a deliberate act or unintentionally by children, but also because of the risk of toxicity from respiratory and dermal exposure. This is particularly important in countries where methylated spirits products are highly likely to contain methanol. Where possible, commercially produced ABHRs should be used and the general public should avoid producing their own ABHRs. If commercially produced products are not available, organisations with the facilities to produce ABHRs should continue their laudable efforts, but whilst adhering to the WHO guidance on formulations.

## Data Availability

Data sharing is not applicable to this article as no datasets were generated or analysed during the current study.

## Abbreviations

WHO: World Health Organization
ABHR: Alcohol-based hand rub
USA: United States of America
UK: United Kingdom
TGA: Therapeutic Goods Administration
